# Supplementary material for: Vps11 and Vps18 of Vps-C membrane traffic complexes are E3 ubiquitin ligases and fine-tune signalling
Source: Nat Commun. 2019 Apr 23;10:1833. doi: 10.1038/s41467-019-09800-y (PMC6478910; doi:10.1038/s41467-019-09800-y)
Supplement: Supplementary file 1 — Supplementary Information [file 41467_2019_9800_MOESM1_ESM.pdf]

Supplementary Information

Vps11 and Vps18 of Vps-C membrane traffic complexes are E3 ubiquitin ligases and fine-tune signalling

Segala et al.

Supplementary Fig. 1

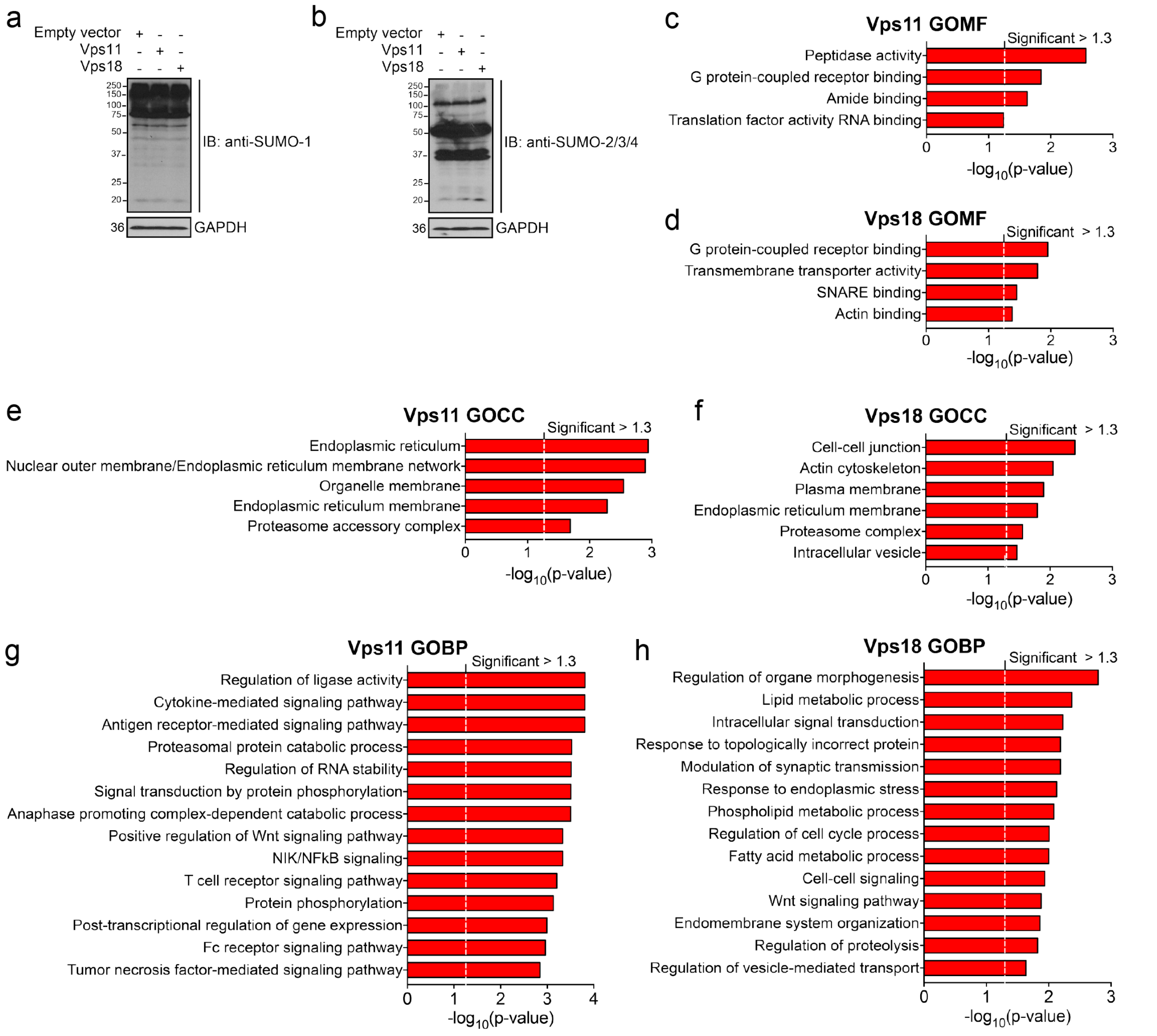

Supplementary Fig. 1 Sumo control experiments and GO analyses. **a, b** *In vivo* sumoylation assay with HEK293T cells overexpressing Vps11/18. Sumoylated proteins were probed for with either a SUMO-1 (panel **a**) or a SUMO-2/3/4 antibody (panel **b**). **c-h** Bar plots showing the -$\log_{10}$(p-value) of GO terms associated with proteins whose ubiquitination significantly changes (-$\log_{10}$(p-value) > 1.3) upon Vps11/18 overexpression. A GSEA was performed on the differentially ubiquitinated sites shared by both Vps11- and Vps18- overexpressing cells compared to the negative control; the Cytoscape plugin Enrichment Map was used to calculate the -$\log_{10}$(p-value) for each GO term. The GO classes were separated into three major categories: GOMF (GO molecular function) (panels **c** and **d**), GOCC (GO cellular component) (panels **e** and **f**), and GOBP (GO biological process) (panels **g** and **h**). Source data are provided as a Source Data file.

Supplementary Fig. 2

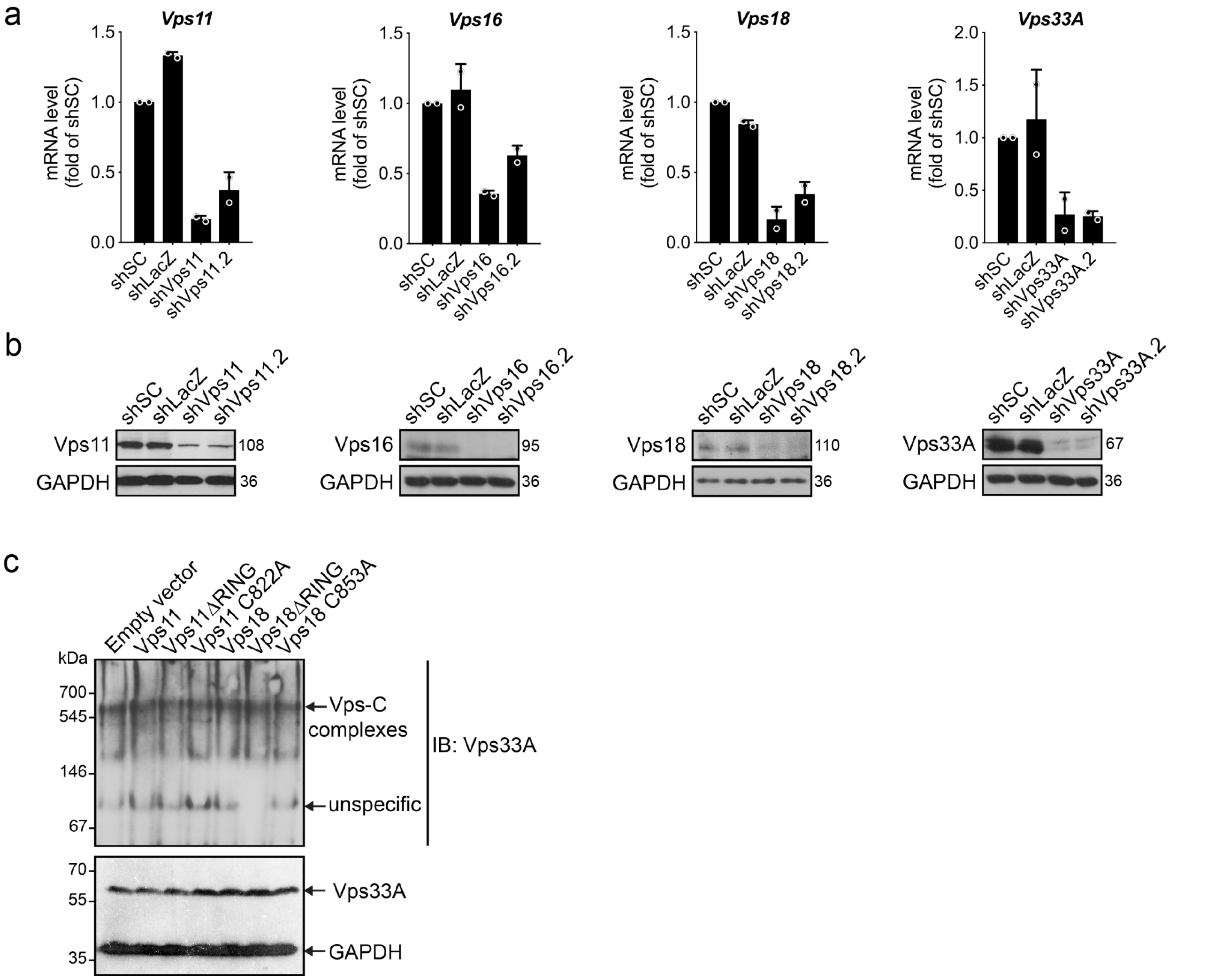

Supplementary Fig. 2 A variety of control and complementary experiments. **a, b** Knock-down of the expression of individual Vps-C core components by the indicated shRNA and determination of their efficiency at the mRNA (panel **a**, mean ± s.e.m. with n = 2 biologically independent experiments with triplicate samples) and protein (panel **b**) levels by quantitative RT-PCR and immunoblotting, respectively. GAPDH was used as a loading and internal control. **c** Immunoblot of a native protein gel of Vps-C complexes of HEK293T cells with overexpression of either Vps11, Vps18 or their corresponding RING domain mutants; the immunoblot was probed with an antibody to Vps33A; in parallel, an immunoblot was performed on the same samples separated under denaturing conditions to determine the total amount of Vps33A. Source data are provided as a Source Data file.

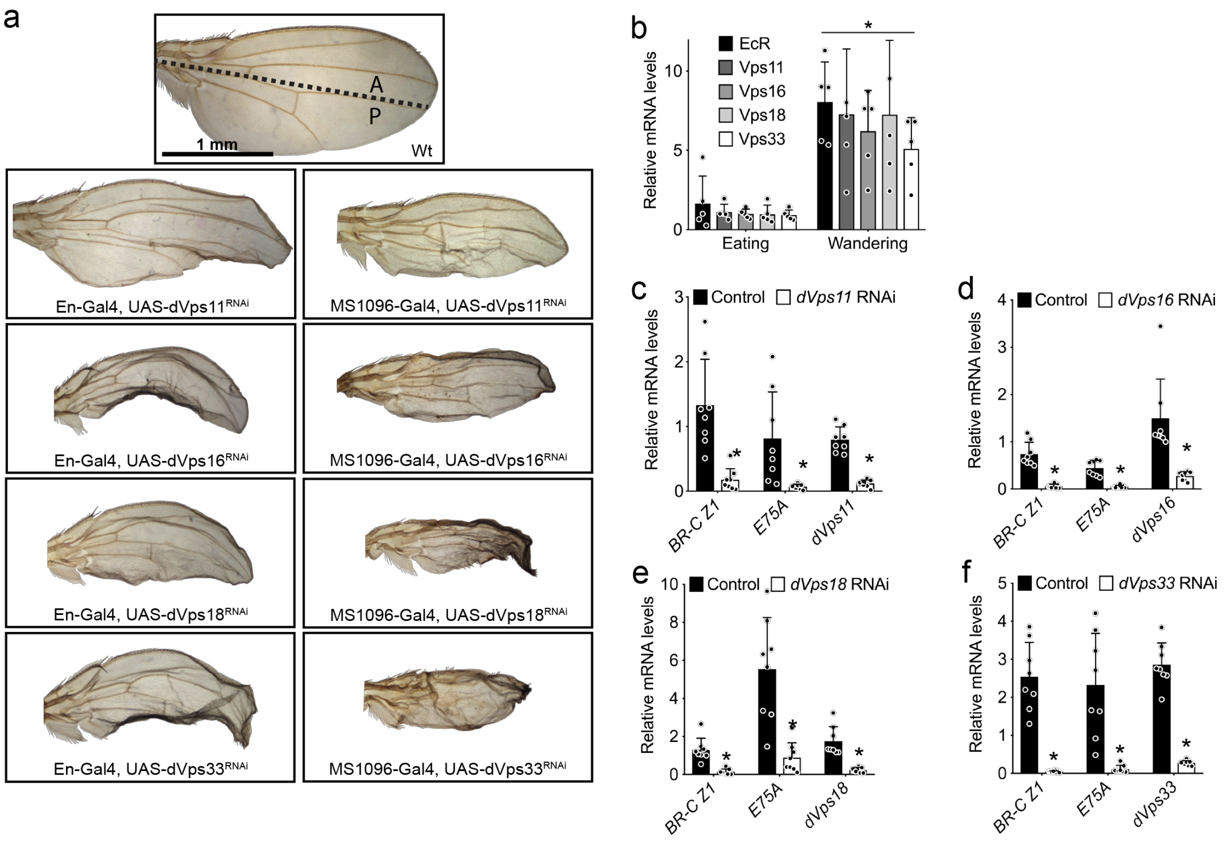

Supplementary Fig. 3 *Drosophila* wing development and ecdysone signalling depend on all components of the Vps-C core. **a** Wings from adult flies with knock-downs of individual Vps-C core components specifically in either the posterior (driver En-Gal4) or the dorsal (driver MS1096-Gal4) compartments. At the top, a wing from a wild-type adult fly with the dotted line delimiting the anterior (A) and posterior (P) compartments. The other micrographs present wings from flies where either the drivers En-Gal4 or MS1096 were used to knock down individual Vps-C core components with the gene-specific UAS-RNAi constructs. Scale bar indicates 1 mm. **b** Quantitative RT-PCR analysis of the mRNA levels of the Vps-C core components and the EcR in early third instar larvae (eating) and late third instar larvae (wandering). The bar graphs show the averages ± s.e.m. of the mRNA levels for n = 5 biologically independent larvae. The asterisk indicates that all the differences of expression between eating and wandering stages were significant with p-values < 0.05. **c-f** Quantitative RT-PCR analysis of the mRNA levels of the EcR target genes BR-C Z1 and E75A and the targeted Vps-C core components in late third instar larvae with a knock-down of either dVps11 (**c**), dVps16 (**d**), dVps18 (**e**) or dVps33 (**f**). The bar graphs show the averages ± s.e.m. of the mRNA levels for n = 8 biologically independent larvae. For panels **c-f**, asterisks indicate significant differences with the corresponding negative controls with p-values < 0.05. Statistical significance was determined with unpaired and two-sided Student's t-tests. Source data are provided as a Source Data file.

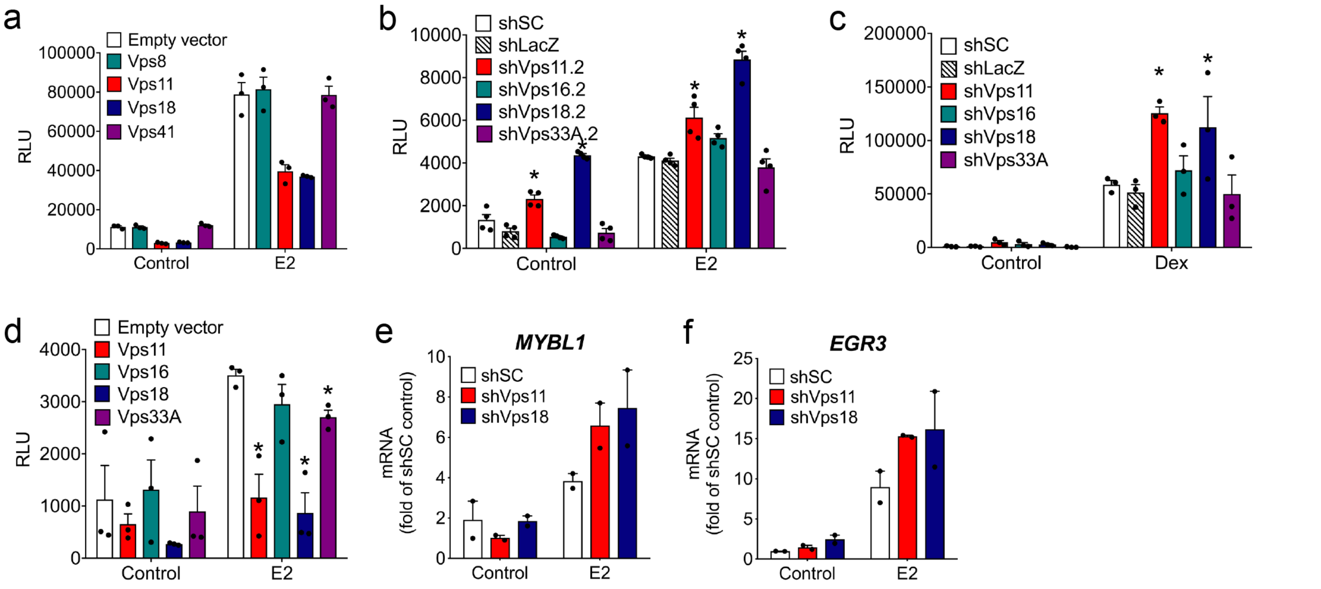

Supplementary Fig. 4 Additional control and complementary experiments. **a-d** Luciferase reporter assays for ERα (panels **a, b, d**) or GR (panel **c**) with HEK293T (panels **a-c**) or MDA-MB-134 cells (panel **d**) overexpressing different Vps-C components or the indicated shRNA constructs. Bar graphs show average ± s.e.m. with n≥3 biologically independent experiments **e, f** Quantitative RT-PCR analysis of the mRNA levels of additional ERα target genes with MDA-MB-134 cells infected with viruses for the indicated shRNA constructs. The bar graphs show the averages ± s.e.m. with n = 2 biologically independent experiments with triplicate samples; asterisks indicate significant differences with the corresponding negative controls with p-values < 0.05. Statistical significance was determined with unpaired and two-sided Student’s t-tests. Source data are provided as a Source Data file.

Supplementary Fig. 5

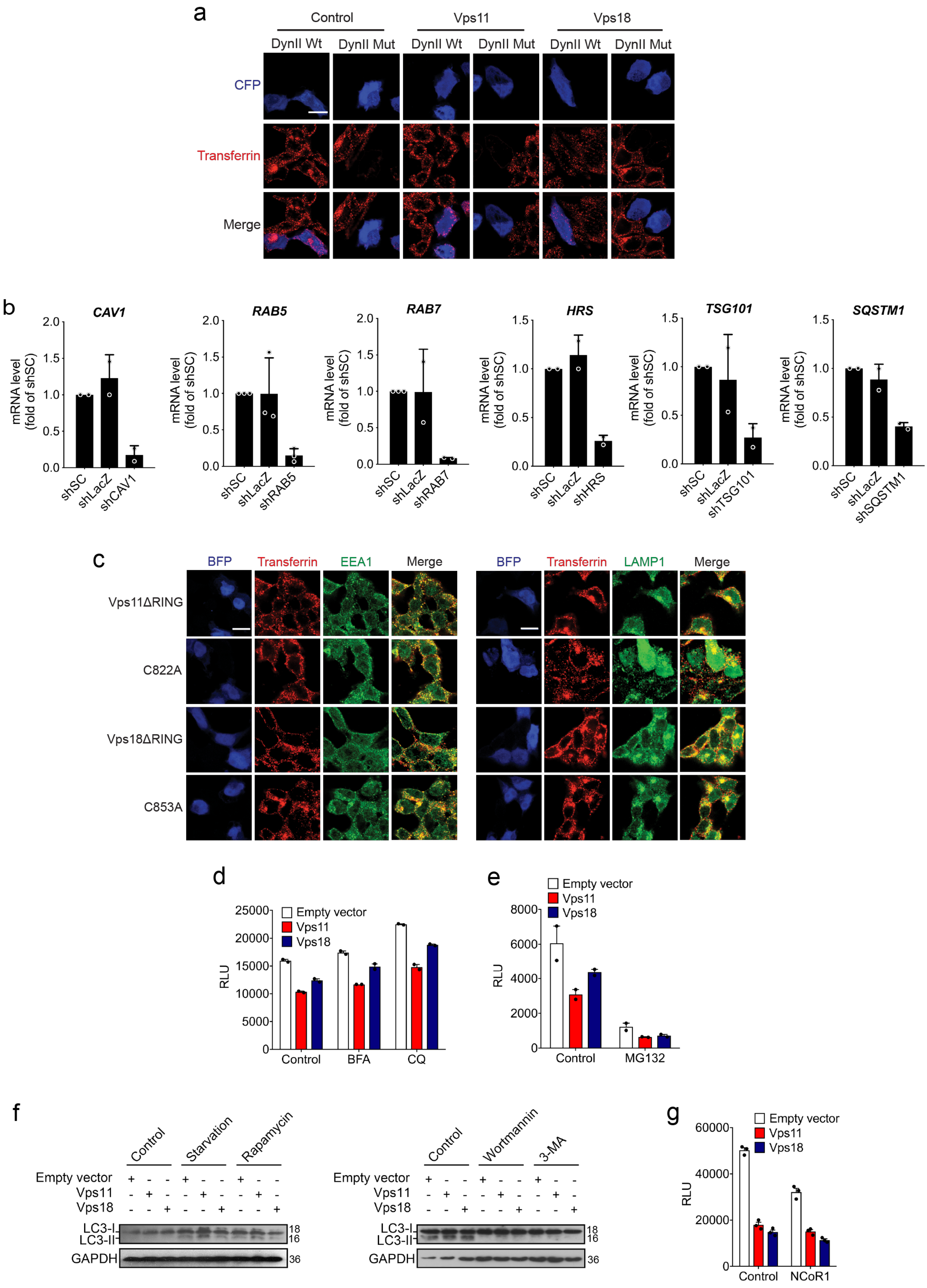

Supplementary Fig. 5 Pathways and factors that are not involved or not affected.
a Transferrin uptake assays in HEK293T cells overexpressing either Vps11, Vps18 or their corresponding RING domain mutants along with CFP. Scale bar indicates 20 µm. **b** Verification of the efficiency of the knock-downs of the expression of several genes involved in intracellular membrane traffic by the indicated shRNAs by quantitative RT-PCR (mean ± s.e.m. with $n \geq 2$ biologically independent experiments with triplicate samples). The GAPDH mRNA was used as an internal control. **c** Transferrin uptake assays with immunostaining of EEA1 and LAMP1 in HEK293T cells overexpressing RING domain mutants of Vps11/18 along with BFP. Scale bar indicates 20 µm. **d, e** Luciferase reporter gene assays for ERα with HEK293T cells overexpressing Vps11/18 and treated with E2 along with brefeldin A (BFA) or chloroquin (CQ) (panel **d**) or MG132 (panel **e**) (mean ± s.e.m. with n = 2 biologically independent experiments with triplicate samples). **f** Immunoblots of extracts from HEK293T cells overexpressing Vps11/18 in combination with the indicated condition or treatments. **g** ERα reporter gene assay with HEK293T cells overexpressing Vps11/18 along with NCoR1 (mean ± s.e.m. with n = 3 biologically independent experiments with triplicate samples); asterisks indicate significant differences with the corresponding negative controls with p-values < 0.05. Statistical significance was determined with unpaired and two-sided Student's t-tests. Source data are provided as a Source Data file.

Supplementary Fig. 6

a

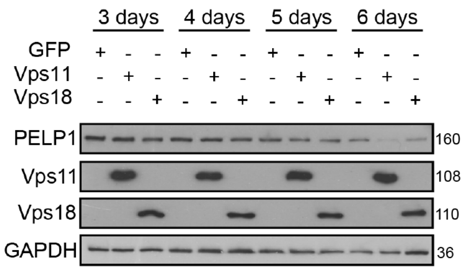

b

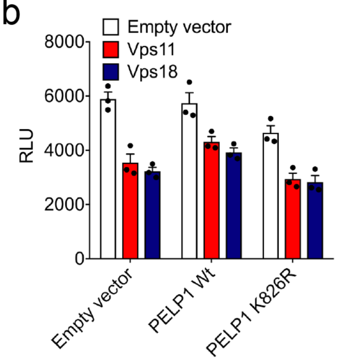

c

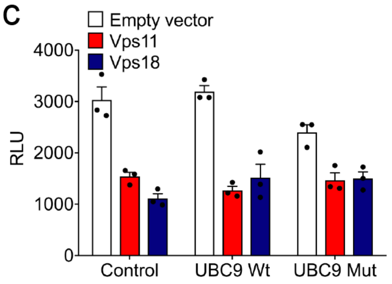

d

Putative Ub site in PELP1	Theoretical tryptic peptide size
K103	7
K200	20
K214	8
K216	10
K255	70
K354	18
K430	46
K474	46
K490	16
K495	6
K496	3
K498	15
K512	16
K541	15
K770	64
K826	224
K994	192
K1075	61
K1100	46
K1121	30

e

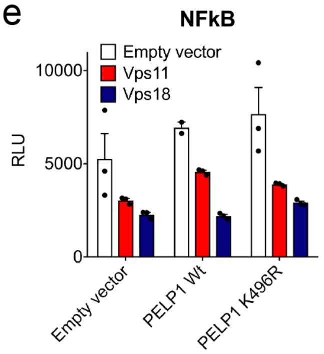

Supplementary Fig. 6 Mapping of ubiquitination site in PELP1 and control experiments. **a** Immunoblot of PELP1 to show the kinetics of its reduction by lentivirus-mediated overexpression of Vps11/Vps18 for the indicated number of days (one representative experiment of three is shown); the protein levels of Vps11/18 are shown to control their expression stability, and GAPDH was used as a loading control. **b, c** Luciferase reporter assays for ERα with HEK293T cells overexpressing Vps11/18 along with wild-type PELP1 or PELP1 K826R (panel **b**) or wild-type or mutant UBC9 (panel **c**); ERα was activated with E2 (panels **b, c**). **d** Table listing the putatively ubiquitinated lysines of PELP1 along with the predicted sizes of the associated tryptic peptides. The lysines highlighted in red were mutated individually for the experiments of Fig. 5f because they correspond to short peptides that could have been missed in the MS analysis. **e** Luciferase reporter gene assay for NFκB with HEK293T cells overexpressing Vps11/18 along with wild-type PELP1 or PELP1 K496R; NFκB was activated with PMA. The bar graphs show the averages ± s.e.m. with n = 3 biologically independent experiments with triplicate samples; asterisks indicate significant differences with the corresponding negative controls with p-values < 0.05. Statistical significance was determined with unpaired and two-sided Student's t-tests. Source data are provided as a Source Data file.

Supplementary Table 1 Details of shRNA constructs. Related to Methods section. All shRNA constructs are based on expression vector pLKO.1.

shRNA	***Target sequence 5'-3'***	***TRCN* / reference / design tool****
**shSC**	CCTAAGGTTAAGTCGCCCTCG	ref. 1
**shLacZ**	CGCTAAATACTGGCAGGCGTT	ref. 2
**shVps11.1**	GACCAAGAGAACAGACACATT	http://sirna.wi.mit.edu
**shVps11.2**	CAGCTGTCCTCAAGCATATTT	http://sirna.wi.mit.edu
**shVps16.1**	GCTCGATATATACTCTGCTTT	http://sirna.wi.mit.edu
**shVps16.2**	GTGCAACAGAAGGATGTCTTT	http://sirna.wi.mit.edu
**shVps18.1**	GGAGAGCATTAAACTGTCTTT	http://sirna.wi.mit.edu
**shVps18.2**	GGGTGAGAAAGTGATGCTATT	http://sirna.wi.mit.edu
**shVps33A.1**	CCACCACTAAACTAATGAATT	http://sirna.wi.mit.edu
**shVps33A.2**	GGGTCAGCTTCTTAAATTATT	http://sirna.wi.mit.edu
**shCav1**	CCACCTTCACTGTGACGAAAT	TRCN0000350508
**shRab5**	GCAGCCTTCCTTTCCAAAGTT	TRCN0000011215
**shRab7**	ACGTAGGCCTTCAACACAATT	TRCN0000381534
**shHRS**	CTCACGTCCGGAGTAACACTA	TRCN0000380920
**shTSG101**	GGTGCCCAGAATAAGTTATTT	http://sirna.wi.mit.edu
**shSQSTM1**	CCTCTGGGCATTGAAGTTGAT	TRCN0000007237

* TRCN, the RNA consortium number (https://www.broadinstitute.org/rnai/trc).

** For sources other than the TRC database or published sequences, shRNAs were designed with an online tool at http://sirna.wi.mit.edu with accession / GI numbers as inputs.

Supplementary Table 2 Primer sequences for RT-PCR. Related to Method section.

mRNA	*Forward primer 5'-3'*	*Reverse primer 5'-3'*
BCL2	GCCCTGTGGATGACTGAGTA	GCAGAGTCTTCAGAGACAGCC
BR-C Z1	TTGGCAGTGGCAGCAACAACAACA	GTGGTGCTTGATCGTACTGAAGTC
CAV1	GTCAACCGCGACCCTAAACA	GAAGCTGGCCTTCCAAATGC
CCND1	AACAGATCATCCGCAAACACG	GGAAGTTGTTGGGGCTCCTC
CXCL12	AACACTCCAAACTGTGCCCT	AGTGGGTCTAGCGGAAAGTC
dGAPDH	CGTTCATGCCACCACCGCTA	CCACGTCCATCACGCCACAA
dVps11	CAATGTTGGTTGTGGGACGG	AGGTAATGGCCTACCCAAGC
dVps16	AGAGCTTGCTTAGGACGGC	CCGCAAGGTGTTAAGAACCC
dVps18	CCTCGACAAGGGTCAGTACG	AGCGTAATAATCGGCTGCCA
dVps33	AGCAACCTGGAGAAATCGGG	TGAGGTGCAGCGTCTTTCG
E75A	AATTCGCGGCGTGATCGACTT	AGCAACTTGGCCAGGAACTCG
EcR	ACTCCAGCCACAGATTCAACCACA	CATGTATTCGCTGCTCGTACTGAC
EGR3	CCGGTGACCATGAGCAGTTTG	GTTGGGCTTCTCGTTGGTCA
GAPDH	GCACAACAGGAAGAGAGAGACC	AGGGGAGATTCAGTGTGGTG
GREB1	ATATGCAAGGCCAAGCCAGT	CTCGTATGCCCGTGAGACAG
HRS	GAGACAGAAGTCCACGTACACT	CGGTTGAGATACCGTGCGAG
MYBL1	ATGTTCAGCCTACTTCTGCCTTT	CCAGACCAGCTAGAAAAACTTCC
RAB5	ACCACCACCGCCATAGATAC	TTCCAGAATTCAAGGGGCCA
RAB7	CCAAGGAGGCCATCAACGTG	TGTACAGCTCCACCTCCGTT
SQSTM1	AAGCCGGGTGGGAATGTTG	CCTGAACAGTTATCCGACTCCAT
TSG101	CCATATCCTGCCACAACAAGT	TCACTGACCGCAGAGATGAGA
VPS11	CAAGCCTACAAACTACGGGTG	GAGTGCAGAGTGGATTGCCA
VPS16	ATGCACCCGAGAGCATCCA	CTGTACTTCAGGAAGGCGCA
VPS18	CAAGGCAAATGAGCCCAACC	GCTAGTGGCCGTACCTTCTG
VPS33A	CCTTTGGCCTGATTGCACAG	AAATCTCTCGTTGGGCCTCG

Supplementary references

1. Sarbassov, D. D., Guertin, D. A., Ali, S. M. & Sabatini, D. M. Phosphorylation and regulation of Akt/PKB by the rictor-mTOR complex. *Science* **307**, 1098-1101 (2005).
2. Rosenbluh, J. *et al.* β-catenin-driven cancers require a YAP1 transcriptional complex for survival and tumorigenesis. *Cell* **151**, 1457-1473 (2012).
