# Supplementary material for: Vps11 and Vps18 of Vps-C membrane traffic complexes are E3 ubiquitin ligases and fine-tune signalling
Source: Nat Commun. 2019 Apr 23;10:1833. doi: 10.1038/s41467-019-09800-y (PMC6478910; doi:10.1038/s41467-019-09800-y)
Supplement: Supplementary file 3 — Description of Additional Supplementary Files [file 41467_2019_9800_MOESM3_ESM.docx]

**Description of Supplementary Files**

**File Name:** Supplementary Data 1

**Description:** Ubiquitinated proteins differentially regulated by Vps11/18 with the log2 fold change of protein ubiquitination when Vps11/18 are overexpressed compared to cells transfected with the empty vector.
