# Supplementary material for: Vps11 and Vps18 of Vps-C membrane traffic complexes are E3 ubiquitin ligases and fine-tune signalling
Source: Nat Commun. 2019 Apr 23;10:1833. doi: 10.1038/s41467-019-09800-y (PMC6478910; doi:10.1038/s41467-019-09800-y)
Supplement: Supplementary file 5 — Reporting Summary [file 41467_2019_9800_MOESM5_ESM.pdf]

nature research

Corresponding author(s): Didier Picard

Reporting Summary

Nature Research wishes to improve the reproducibility of the work that we publish. This form provides structure for consistency and transparency in reporting. For further information on Nature Research policies, see Authors & Referees and the Editorial Policy Checklist.

Statistical parameters

When statistical analyses are reported, confirm that the following items are present in the relevant location (e.g. figure legend, table legend, main text, or Methods section).

n/a	Confirmed	
☒	☐	The exact sample size (*n*) for each experimental group/condition, given as a discrete number and unit of measurement
☐	☒	An indication of whether measurements were taken from distinct samples or whether the same sample was measured repeatedly
☐	☒	The statistical test(s) used AND whether they are one- or two-sided<br>*Only common tests should be described solely by name; describe more complex techniques in the Methods section.*
☒	☐	A description of all covariates tested
☒	☐	A description of any assumptions or corrections, such as tests of normality and adjustment for multiple comparisons
☐	☒	A full description of the statistics including central tendency (e.g. means) or other basic estimates (e.g. regression coefficient) AND variation (e.g. standard deviation) or associated estimates of uncertainty (e.g. confidence intervals)
☒	☐	For null hypothesis testing, the test statistic (e.g. *F*, *t*, *r*) with confidence intervals, effect sizes, degrees of freedom and *P* value noted<br>*Give P values as exact values whenever suitable.*
☒	☐	For Bayesian analysis, information on the choice of priors and Markov chain Monte Carlo settings
☒	☐	For hierarchical and complex designs, identification of the appropriate level for tests and full reporting of outcomes
☒	☐	Estimates of effect sizes (e.g. Cohen's *d*, Pearson's *r*), indicating how they were calculated
☐	☒	Clearly defined error bars<br>*State explicitly what error bars represent (e.g. SD, SE, CI)*

Our web collection on statistics for biologists may be useful.

Software and code

Policy information about availability of computer code

Data collection	Mass spectrometry data acquisition was performed with Xcalibur 2.1 (Thermo Fisher).
Data analysis	Mass spectrometry data were analyzed with MaxQuant 1.5.3.30 followed by filtering steps, statistics and annotation enrichment analyses with Perseus. Gene set enrichment analysis was carried out with GSEA 3.0 (Broad Institute) and the gene ontology study with Enrichment Map from Cytoscape. Interactome was generated with PpiMapBuilder plug-in (https://goo.gl/GusMZG) for Cytoscape.

For manuscripts utilizing custom algorithms or software that are central to the research but not yet described in published literature, software must be made available to editors/reviewers upon request. We strongly encourage code deposition in a community repository (e.g. GitHub). See the Nature Research guidelines for submitting code & software for further information.

Data

Policy information about availability of data

All manuscripts must include a data availability statement. This statement should provide the following information, where applicable:

- Accession codes, unique identifiers, or web links for publicly available datasets
- A list of figures that have associated raw data
- A description of any restrictions on data availability

The mass spectrometry proteomics data have been deposited to the ProteomeXchange Consortium (http://proteomecentral.proteomexchange.org) via the PRIDE partner repository with identifier PXD009178.

Field-specific reporting

Please select the best fit for your research. If you are not sure, read the appropriate sections before making your selection.

☒ Life sciences ☐ Behavioural & social sciences ☐ Ecological, evolutionary & environmental sciences

For a reference copy of the document with all sections, see nature.com/authors/policies/ReportingSummary-flat.pdf

Life sciences study design

All studies must disclose on these points even when the disclosure is negative.

Sample size	No sample size was pre-determined. Three to more independent results were used to perform statistical analyses.
Data exclusions	No data were excluded from analysis.
Replication	Experiments in the article were reliably reproduced, replication were described in the figure legends.
Randomization	No formal randomization techniques was used. No animals and/or human research participants were involved.
Blinding	Investigators were not blinded to group allocation during data collection and/or analysis.

Reporting for specific materials, systems and methods

Materials & experimental systems

n/a	Involved in the study
☐	☒ Unique biological materials
☐	☒ Antibodies
☐	☒ Eukaryotic cell lines
☒	☐ Palaeontology
☒	☐ Animals and other organisms
☒	☐ Human research participants

Methods

n/a	Involved in the study
☒	☐ ChIP-seq
☒	☐ Flow cytometry
☒	☐ MRI-based neuroimaging

Unique biological materials

Policy information about availability of materials

Obtaining unique materials	Several plasmids were built in this study and are available from the authors upon request

Antibodies

Antibodies used	Rabbit anti-ERα antibody, clone HC-20, Santa Cruz Biotechnologies, sc-543, discontinued<br>Rabbit anti-pERα Ser118 antibody, clone Ser118, Santa Cruz Biotechnologies, sc-12915-R, discontinued<br>Mouse anti-ERK1/2 antibody, clone C-9, Santa Cruz Biotechnologies, sc-514302<br>Mouse anti-pERK antibody, clone E-4, Santa Cruz Biotechnologies, sc-7383

Mouse anti-Vps11 antibody, clone S-38, Santa Cruz Biotechnologies, sc-100893
Mouse anti-SUMO-2/3/4 antibody, clone C-3, Santa Cruz Biotechnologies, sc-393144
Goat anti-Vps16 antibody, clone C-17, Santa Cruz Biotechnologies, sc-86939, discontinued
Rabbit anti-PELP1 antibody, Bethyl Laboratories, A300-180A
Rabbit anti-BCAR1/p130Cas antibody, Bethyl Laboratories, A301-667A
Mouse anti-GAPDH antibody, clone 6C5, Abcam, ab8245
Mouse anti-HA.11 antibody, clone 16B12, Biolegend, MMS-101P
Rabbit anti-Vps33A antibody, Thermo Fisher Scientific, PA545268
Rabbit anti-pERα Ser167 antibody, Thermo Fisher Scientific, PA537570
Rabbit anti-pERα Tyr537 antibody, Thermo Fisher Scientific, PA537571
Mouse anti-Vps18 antibody, Thermo Fisher Scientific, MA522391
Rabbit anti-PKA substrates (P-(S/T)) antibody, Cell Signaling Technology, 9621
Rabbit anti-pSrc Tyr416 antibody, clone D49G4, Cell Signaling Technology, 6943
Rabbit anti-PKA antibody, Upstate, 06-903
Mouse anti-Src antibody, clone GD11, Millipore, 05-184
Rabbit anti-SUMO-1 antibody, Alexis biochemicals, BML-PW0505A
Rabbit anti-EEA1, Enzo, ALX-210-239
Mouse anti-LAMP1, BD PharMingen, H4A3

Validation

- Rabbit anti-ERα antibody, clone HC-20, validated by manufacturer for western blot application in human cells by using recombinant ERα.
- Rabbit anti-pERα Ser118 antibody, clone Ser118, validated by manufacturer for western blot application in human cells by treating with growth factor -/+ phosphatase.
- Mouse anti-ERK1/2 antibody, clone C-9, validated by manufacturer for western blot application in human whole cell lysates.
- Mouse anti-pERK antibody, clone E-4, validated by manufacturer for western blot application in human whole cell lysates.
- Mouse anti-Vps11 antibody, clone S-38, validated by manufacturer for western blot application in human cell lysates.
- Mouse anti-SUMO-2/3/4 antibody, clone C-3, validated by manufacturer for western blot application in human whole cell lysates
- Goat anti-Vps16 antibody, clone C-17, validated by manufacturer for western blot application in human whole cell lysates
- Rabbit anti-PELP1 antibody, validated by manufacturer for western blot and immunoprecipitation applications in human whole cell lysates
- Rabbit anti-BCAR1/p130Cas antibody, validated by manufacturer for western blot application in human whole cell lysates.
- Mouse anti-GAPDH antibody, clone 6C5, validated by manufacturer for western blot application in human whole cell lysates
- Mouse anti-HA.11 antibody, clone 16B12, validated by manufacturer for western blot and immunoprecipitation applications in human whole cell lysates
- Rabbit anti-Vps33A antibody, validated by manufacturer for western blot application in human whole cell lysates
- Rabbit anti-pERα Ser167 antibody, validated by manufacturer for western blot application in human whole cell lysates
- Rabbit anti-pERα Tyr537 antibody, validated by manufacturer for western blot application in human whole cell lysates
- Mouse anti-Vps18 antibody, validated by manufacturer for western blot application in human whole cell lysates
- Rabbit anti-PKA substrates (P-(S/T)) antibody, validated by manufacturer for western blot application in human whole cell lysates
- Rabbit anti-pSrc Tyr416 antibody, clone D49G4, validated by manufacturer for western blot application in human whole cell lysates
- Rabbit anti-PKA antibody, validated by manufacturer for western blot application in human whole cell lysates
- Mouse anti-Src antibody, clone GD11, validated by manufacturer for western blot and immunoprecipitation applications in human whole cell lysates
- Rabbit anti-SUMO-1 antibody, validated by manufacturer for western blot application in human whole cell lysates
- Rabbit anti-EEA1, validated by Mai et al., Nat Chem 2017 for immunofluorescence application in human cells.
- Mouse anti-LAMP1, validated by Mai et al., Nat Chem 2017 for immunofluorescence application in human cells.

Eukaryotic cell lines

Policy information about cell lines

Cell line source(s)

HEK293T cells were purchased from the American Type Culture Collection (ATCC), MDA-MB-134 cells were a gift from Wilbert Zwart.

Authentication

Cell lines were not authenticated by ourselves.

Mycoplasma contamination

All cell lines were tested to be mycoplasma negative.

Commonly misidentified lines
(See ICLAC register)

None of the cell lines used in this study were found in the commonly misidentified cell lines database.
